# Single-unit activities during epileptic discharges in the human hippocampal formation

**DOI:** 10.3389/fncom.2013.00140

**Published:** 2013-10-18

**Authors:** Catalina Alvarado-Rojas, Katia Lehongre, Juliana Bagdasaryan, Anatol Bragin, Richard Staba, Jerome Engel, Vincent Navarro, Michel Le Van Quyen

**Affiliations:** ^1^Centre de Recherche de l'Institut du Cerveau et de la Moelle Epinière, INSERM UMRS 975 - CNRS UMR 7225, Hôpital de la Pitié-SalpêtrièreParis, France; ^2^Université Pierre et Marie CurieParis, France; ^3^Department of Neurology, David Geffen School of Medicine at UCLALos Angeles, CA, USA; ^4^Epilepsy Unit, Groupe Hospitalier Pitié-SalpêtrièreParis, France

**Keywords:** interictal epileptiform discharges, microelectrode recordings, multiunit activity, temporal lobe epilepsy, spike synchronization

## Abstract

Between seizures the brain of patients with epilepsy generates pathological patterns of synchronous activity, designated as interictal epileptiform discharges (ID). Using microelectrodes in the hippocampal formations of 8 patients with drug-resistant temporal lobe epilepsy, we studied ID by simultaneously analyzing action potentials from individual neurons and the local field potentials (LFPs) generated by the surrounding neuronal network. We found that ~30% of the units increased their firing rate during ID and 40% showed a decrease during the post-ID period. Surprisingly, 30% of units showed either an increase or decrease in firing rates several hundred of milliseconds before the ID. In 4 patients, this pre-ID neuronal firing was correlated with field high-frequency oscillations at 40–120 Hz. Finally, we observed that only a very small subset of cells showed significant coincident firing before or during ID. Taken together, we suggested that, in contrast to traditional views, ID are generated by a sparse neuronal network and followed a heterogeneous synchronization process initiated over several hundreds of milliseconds before the paroxysmal discharges.

## Introduction

**Figure 1:**
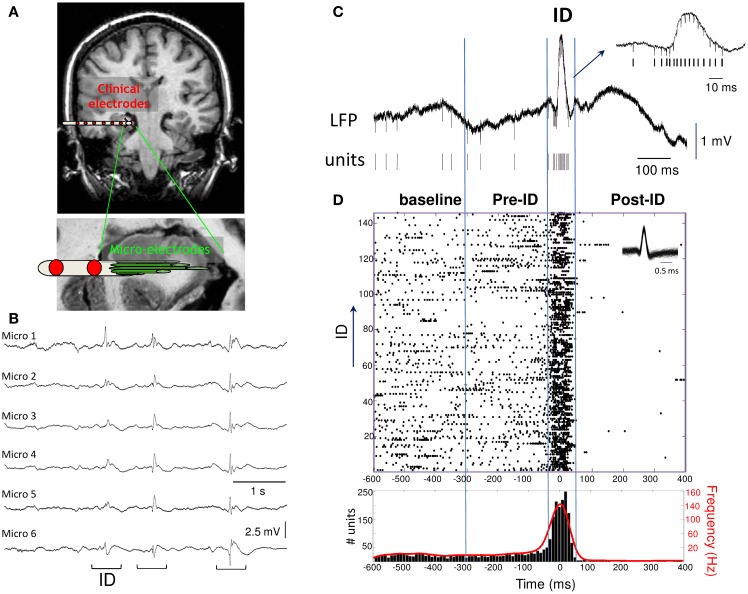
**(A)** Macro- and micro-electrodes superimposed on a magnetic resonance imaging scan. Nine microwires (40 μm diameter) extend beyond the tip of each macro-electrode and record the hippocampal formation. **(B)** Interictal discharges (ID) recorded with microelectrode local field potentials from adjacent electrodes in the hippocampus of a patient. **(C)** Example of wide-band recording of an ID event with the corresponding extracted single unit activities. **(D)** Raster plot and peri-event histogram (bin size, 10 ms) of the single unit activity shown above. Note the strong changes in the firing rate and instantaneous frequency (red) during the ID.

Synchronization of local and distributed neuronal assemblies is thought to underlie fundamental brain processes such as perception, learning, and cognition (Varela et al., 2001). In neurological diseases, neuronal synchrony can be altered and in epilepsy may play an important role in enhanced cellular excitability (Jasper and Penfield, 1954). Besides ictal events or seizures, interictal discharges (ID) are a typical signature of abnormal neuronal synchronization, seen spontaneously between seizures in scalp and intracranial EEG. They are used as a clinical indicator for the location of the epileptogenic zone, the region that generates seizures. Furthermore, it is believed that this region contains both, the seizure onset zone and the surrounding “irritative zone,” which generates ID and limits with normal tissue (Talairach and Bancaud, 1966). These transient epileptic synchronization events are characterized by a large-amplitude, rapid component lasting 50–100 ms that is usually followed by a slow wave of 200–500 ms duration (de Curtis and Avanzini, 2001). In some cases, they are associated with an oscillation in the high frequency range greater than 40 Hz (Bragin et al., 1999; Jacobs et al., 2011; Le Van Quyen, 2012). Despite their fundamental importance in diagnosing and treating epilepsy, little is known about the neurophysiological mechanisms generating these events in the human brain. Experimental work on animals and human tissue propose the paroxysmal depolarization shift (PDS) as the cellular correlate of ID (Prince and Wong, 1981; Avoli and Williamson, 1996). This event is defined as a burst of action potentials on a large depolarization, followed by a longer hyperpolarization. However, *in vivo* human evidence is scarce, because of the limited opportunities to study the behavior of single neurons in human subjects. To overcome this difficulty, epilepsy patients suitable for surgical treatment are sometimes studied with intracranial depth electrodes in order to record EEG activity from deep cortical structures and accurately identify the regions originating seizures. Using depth electrodes specially adapted with microelectrodes (Fried et al., 1997; Figure 1A), ID can be studied by simultaneously recording action potentials from individual neurons and the local field potentials (LFP). Studies using microelectrode technology, have reported a variable and complex relation between ID and the activity of individual neurons, more heterogeneous than simple PDS (Babb et al., 1973; Wyler et al., 1982; Ulbert et al., 2004; Keller et al., 2010; Alarcon et al., 2012). In particular, a large diversity of neuronal response were found including an increase or decrease in their firing rates or even changes in firing that precede the defining interictal discharge itself. Most of these studies were performed on patients with neocortical epilepsy that exhibit a wide range of heterogeneity. In the present work, we recorded ID in the hippocampal formation of 8 patients with drug-resistant mesial temporal lobe epilepsy. Our objective is to describe firing patterns and neuronal synchronization of single-unit activities during spontaneous IDs.

## Materials and methods

### Database

Subjects were 8 patients [two female, mean age ± standard deviation (SD) 36.3 ± 10.5 years] with pharmacologically intractable temporal lobe epilepsy who were implanted with 8–14 intracranial depth electrodes in order to localize epileptogenic regions for possible resection. The placement of the electrodes was determined exclusively by clinical criteria (Fried et al., 1999). Extending beyond the tip of each electrode were nine Pt-Ir microwires (40 μm diameter) with inter-tip spacing of 500 μm, eight active recording channels and one reference. Each microwire was sampled at 28 kHz (Cheetah recording system, Neuralynx Inc., Tucson, AZ). Spatial localizations were determined on the basis of postimplant computed tomography scans coregistered with preimplant 1.5T MRI scans. Our results are based on microelectrode recordings located in the anterior hippocampus (*n* = 40 channels in 5 patients) and entorhinal cortex (*n* = 24 channels in 3 patients). The recording states were quiet wakefulness and slow waves sleep (stages 1–4). All studies conformed to the guidelines of the Medical Institutional Review Board at University of California, Los Angeles.

### Spike sorting

In order to detect single-units, all channels were high-pass filtered at 300 Hz and were visually examined for the presence of unit activities. In those microwires with clear unit activities, we performed spike detection (>4:1 signal to noise ratio) to obtain multi-unit activities (MUA). Single-unit activities were extracted with spike sorting using KlustaKwik 1.7 program (Software: http://klustakwik.sourceforge.net/; Harris et al., 2000) which employs the 10 principal components of the spike shape and an unsupervised Conditional Expectation Maximization (CEM) clustering algorithm (Hazan et al., 2006). After automatic clustering, the clusters containing non-spike waveforms were visually deleted and then the units were further isolated using a manual cluster cutting method. Only units with clear boundaries and less than 0.5% of spike intervals within a 1 ms refractory period are included in the present analysis. Typically we isolated 1 or 2 distinct neurons from each microwire, but in several cases we observed up to 4 distinct neurons from a single microwire. The instantaneous spike frequency was measured by convolving the timing of each unit with a Gaussian function of standard deviation of 20 ms (*Ts* = 1 ms), set close to the modal interspike interval (Le Van Quyen et al., 2008, 2010). This operation leads to an analog trace of the instantaneous firing rate (Paulin, 1996).

### Oscillation analysis

LFP are complementary to action potential information and have shown prominent oscillatory activity within the high-frequency frequency range from 40 to 300 Hz (Worrell et al., 2012). A wavelet time-frequency analysis was used to determine precisely the mean frequency, maximum amplitude and onset and offset of these LFP oscillations. The advantage of the wavelet analysis lies in the fact that the time resolution is variable with frequency, so that high frequencies have a sharper time resolution (Le Van Quyen and Bragin, 2007). The Complex Morlet wavelet was here applied that uses a wave-like scalable function that is well-localized in both time and frequency:

$$\Psi_{\tau ,f}(u)=\sqrt{f}\exp(j2\pi f(u-\tau ))\exp\text{​​}\left(-\frac{{\left(u-\tau \right)}^{2}}{2\sigma^{2}}\right).$$

This wavelet represents the product of a sinusoidal wave at frequency *f*, with a Gaussian function centered at time τ, with a standard deviation σ proportional to the inverse of *f*. The wavelet coefficients of a signal *x(t)* as a function of time (τ) and frequency (*f*) are defined as: *W*(τ, *f*) = ∫^+∞^_−∞_ *x*(*u*) Ψ_τ,*f*_(*u*)*du*. It depends solely on σ, which sets the number of cycles of the wavelet: *nco* = 6*f*σ. The value *nco* determines the frequency resolution of the analysis by setting the width of the frequency interval for which phase are measured. Here, we chose *nco* = 5. For baseline correction, the average and SD of power were first computed at each frequency of the baseline period. Then, the average baseline power was subtracted from all time windows at each frequency, and the result scaled by 1/SD, yielding baseline-adjusted *Z* scores. Significant increases with respect to baseline activity showed up as positive *Z*-values and tabulated probability values indicate that, for absolute values of *Z* > 3.09, we have *P* < 0.001. The Kolmogorov–Smirnov test was applied to assess the distribution normality of the wavelet coefficients, using a 0.05 probability level.

### Spike synchronization

Different measures exist to detect and quantify synchronization between spike trains (Brown et al., 2004; Kreuz et al., 2007). In this study, we used two complementary techniques: (1) Cross-correlation analysis was performed for cell pairs (Perkel et al., 1967; Amarasingham et al., 2012). To evaluate the significance of the correlation, we used a boot-strap method that accounts for the firing rate changes of the neurons (Hatsopoulos et al., 2003; Grün, 2009). Since the widths of the peaks in the original cross-correlograms were typically in the range of 5–30 ms (Krüger and Mayer, 1990), the spikes were jittered by adding a random value from a normal distribution with a 50-ms SD and 0 mean to the spike times. For each cell pair, 1000 jittered spike trains were created, and the expected cross-correlogram (and 99% confidence interval) was estimated on 1 ms time bins. For any given cell pair where at least one bin in the [1.5 ms, 30 ms] interval exceeded the 99% confidence interval, the interaction was considered significant. (2) A method for identifying statistically conspicuous spike coincidences was implemented to detect the number of quasi-simultaneous appearances of spikes over small coincidence windows, here of 5 ms (Gütig et al., 2002; Quian Quiroga et al., 2002). Their occurrence was then studied in relation to surrogate data generated by dithering the individual, original spike times within a given time interval. Here, each spike in the original data set was randomly and independently jittered on a uniform interval of [−5, +5] ms to form a surrogate dataset. By repeating the procedure 1000 times, the 99.9% confidence interval for each bin (*p* = 0.001) was calculated.

## Results

Microelectrode recordings were selected by an expert electroencephalographer to have very abundant and persistent ID in the hippocampus (5 patients) or entorhinal cortex (3 patients) during quiet wakefulness or slow-wave sleep (recording durations from 10 to 118 min; total recording time: 6 h). All ID were recorded in the epileptic zone and appeared as spatially synchronous patterns emerging at about the same time on the same bundle of microelectrodes (Figure 1B). A standard, threshold-based ID detector was performed to automatically detect, from the LFP, events showing a pointed peak with a large amplitude, large slope and duration of 20–100 ms, appearing at a frequency of 0.07 ± 0.30 Hz (range: 0.01–0.21 Hz). After expert visual confirmation, 862 ID were identified showing a large pattern of morphological characteristics typical for sharp waves, spikes and spike-wave discharges (Niedermeyer, 2005). Events were aligned by the sharpest peak of the discharge (Figure 1C). In order to analyze the patterns of neuronal activity around the discharge, we defined a baseline period (–600 to –300 ms), pre-ID period (–300 to –50 ms), the interictal discharge (–50 to 50 ms), the post-ID period (50–400 ms). The activities of different neurons per microelectrode were identified with a spike sorting algorithm and a total of 75 single units were selected for analysis. To visualize the discharge-related activity of single neurons, peri-stimulus raster plots and timing histograms were constructed for the period of 1 s before and after each event (Figure 1D).

**Figure 2:**
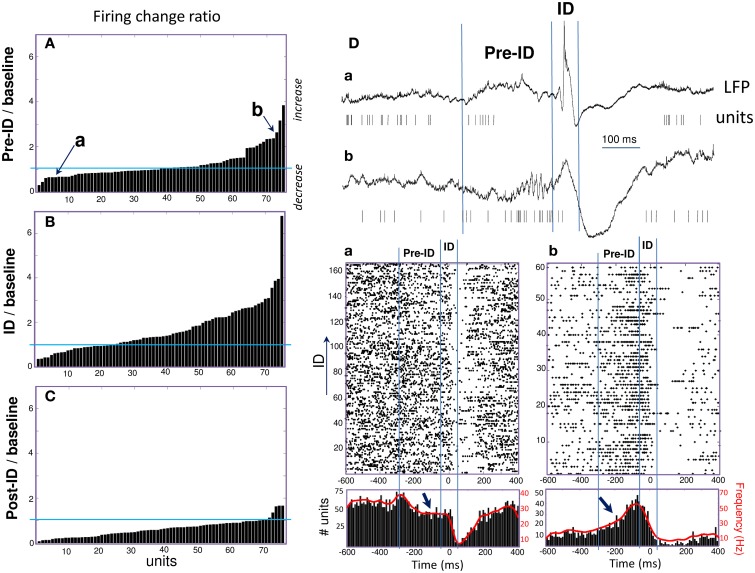
**(A–C)** Peri-ID firing changes of single units defined as the ratio of changes in discharge probability between the baseline and pre-ID, ID or post-ID. **(D)** Examples of two units (Top: raw data; Bottom: Raster plots and peri-event histograms) recorded in different patients and showing significant decrease **(a)** or increase **(b)** during the pre-ID period (see arrows).

**Figure 3:**
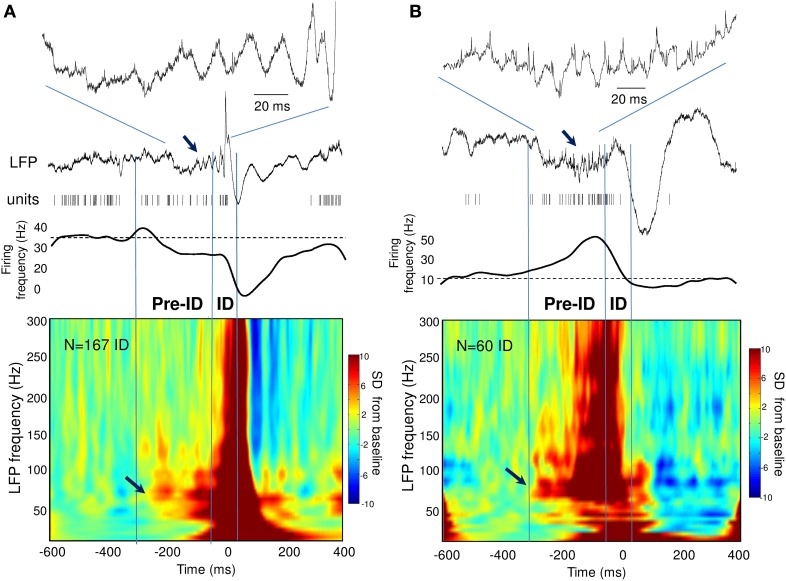
**(A,B)** Time-frequency representations of the LFP around the ID for two patients showing pre-ID changes in neuronal firing. Note the increase in LFP high-frequency oscillations between 40 and 120 Hz preceding the ID and closely correlated with the decrease **(A)** or increase **(B)** in neuronal firing (see arrows).

During the ID period (–50 to 50 ms), we found that around 40% of the recorded units showed some change in firing, whereas 60% remain unchanged. About 32% increased their firing rate more than 2 times during ID relative to baseline epochs [Figure 2B; right-tail *t*-test: *T*_(23)_ = 1.78; *p* = 0.04; an example of a cell can be seen in Figure 1D]. The firing rate of these cells showed a considerable degree of variability (range from 1.4 to 99 Hz) with a mean of 9.4 ± 19.7 Hz during ID (baseline: 2.7 ± 3.1 Hz). During the post-ictal period, 40% of units decreased firing by half [50–400 ms, mean firing rate: 1.8 ± 2.7 Hz and baseline: 7.0 ± 2.7 Hz, left-tail *t*-test: *T*_(29)_ = −3.73; *p* = 4.1·10^−4^, Figure 2C]. In addition to this modulated single unit activity during ID, many units showed a significant change in firing preceding the interictal discharge. From 30% of single units that significantly changed during the pre-ID period, 12% increased [mean firing rate: 10.0 ± 13.5 Hz and baseline: 4.2 ± 5.8 Hz; *T*_(8)_ = −3.45; *p* = 0.004] and 18% decreased [mean firing rate: 2.3 ± 7.0 Hz and baseline: 5.2 ± 11.6 Hz; *T*_(13)_ = −1.64; *p* = 0.06] their firing rate (–300 to –50 ms, Figure 2A; examples are given in Figure 2D). On the corresponding channels, we were interested in the relationship between these pre-ID firing changes and LFP (<300 Hz). Spectral power was performed by using Morlet wavelet analysis (20–300 Hz) and pre-ID changes in LFP were tested for significant increases/decreases from baseline of specific frequency bands (*p* < 0.001). In 4 subjects we observed that pre-ID neuronal firing pattern was correlated with an increase in high-frequency oscillations between 40 and 120 Hz (mean peak from baseline SD: *Z* = 6.1, range from 4.3 to 9.1). Figure 3 shows average time-frequency representations around the ID for the two patients of Figure 2D. Main changes in spectral power can be seen in the LFP preceding the interictal discharge and correlate closely with the increase or decrease in neuronal firing.

**Figure 4:**
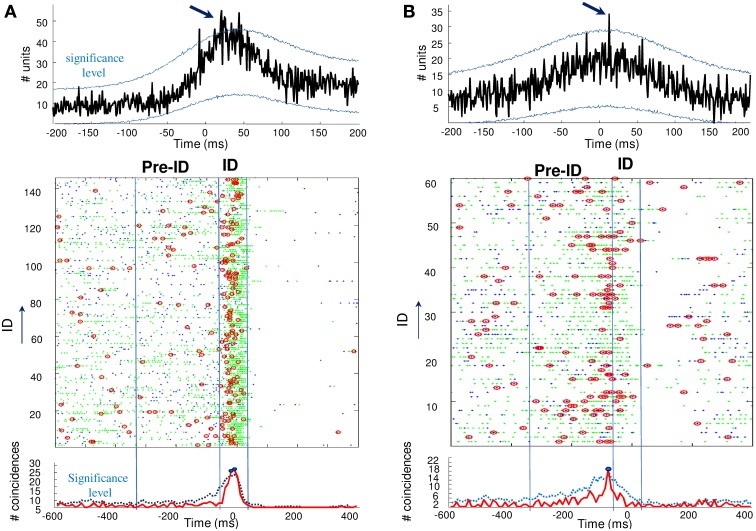
**Top: Cross-correlograms between pairs of units during ID in two patients (A,B)**. The blue lines are the significance levels computed from 1000 jittered spike trains. In both cases, the center peak exceeds the significance level (arrows) and the pairs of units are considered to be significantly correlated. Bottom: Unit synchronizations (red circles) were defined as coincidences between the two units (green and blue points) occurring over a 5-ms interval. Note the significant increase in coincidences during ID **(A)** and the pre-ID period **(B)**, over the statistical threshold defined by a random jitter of the original data.

Finally, we analyzed unit synchronization during ID between pairs of units simultaneously recorded in two different microelectrodes. Because of the inter-tip spacing of 500 μm, the units are assumed to reflect adjacent but different neuronal populations. Two complementary methods have been used to address the synchrony between spike trains. First, analysis of cross-correlograms between pairs of units was performed for each cell pairs that showed a sufficient number of spikes (>100) during ID. The significance of the correlation was obtained by jittering each pair of spike trains and by computing the 99% confidence interval. Of the 120 cross-correlograms constructed, only 5 cross-correlograms (about 4%) had a significant peak that occurred within ±25 ms around the origin, indicating that these neuronal pairs were discharging in a correlated way. Figure 4 (top) illustrates examples of significant peaks in cross-correlograms of two units. In addition to cross-correlation analysis, we also analyzed the overall level of synchronicity from the number of quasi simultaneous appearances of spikes. In order to not overestimate the number of random synchronous spikes due to the elevated firing rate, we used jitter techniques to infer millisecond-precise temporal synchrony (Hatsopoulos et al., 2003). Here, spikes of one of the pairs of neurons were time jittered by ±5 ms to generate jittered peri-stimulus raster plots of unit coincidence that could be used to assess the statistical significance of bin fluctuations in the non-jittered spike series. Because the jittered data sets preserve firing rates on timescales much broader than that of the jitter interval (in this case, 5 ms), the overall effect of the analysis is to identify those pairs that showed excessive co-firing at short latencies that cannot be accounted for by firing rates varying at timescales of tens of milliseconds. Despite the strong increase in about 30% of the recorded units during ID, only a very small subset of cells (18 of 120 analyzed pairs, about 15%) showed significant coincident firing before or during ID. For two patients, Figure 4 (bottom) illustrates pairs of units that showed significant coincident firing (*p* = 0.001) during ID (A) and the pre-ID period (B).

## Discussion

We found that a large subset of the recorded units showed significant changes in firing in or around ID in the hippocampal formation of patients with mesial temporal epilepsy. Around 30% of the unit increased their firing rate during ID while 40% showed a decrease during the post-ID period. This percentage of modulated neurons agrees with that described by Wyler et al. (1982), who found that 44% of recorded neurons showed primarily an increase in firing rate near the interictal discharge peak. Surprisingly, a subset of 30% of units showed significant firing rate variations several hundred of milliseconds before the ID. In a few patients, we observed that this neuronal firing pattern was related with elevated LFP oscillations at 40–120 Hz. Finally, based on two statistical methods that identify spike synchronization, we found that only a very small subset of cells showed significant coincident firing before or during ID.

Our observations of neuronal firing during the interictal discharge are consistent with the paroxysmal depolarizing shift (PDS) mechanism—a large depolarization phase followed by a long hyperpolarization—that have been studied in animal models of epilepsy (Matsumoto and Marsan, 1964; Prince, 1968). The first part of the depolarization phase is believed to be generated by intrinsic membrane conductance (de Curtis et al., 1999), and the later from feedback recurrent synaptic excitation mediated by AMPA and NMDA receptor subtypes, and glutamate receptor-coupled calcium conductances (Traub et al., 1993). Thus, PDS has been shown to be the result of giant excitatory postsynaptic potentials. The PDS is usually followed by a hyperpolarization, which represents GABA-mediated recurrent inhibition, as well as Ca^2+^-dependent K^+^ currents. Interestingly, consistent with *in vitro* studies on hippocampal slices from human patients with temporal lobe epilepsy (Cohen et al., 2002; Wittner et al., 2009), the presence of a similar suppression of unit activities in our *in vivo* data suggests that IDs can occur in cortical regions maintaining substantial inhibitory function.

However, in contrast to simple models of PDS and in line with other observations in human epileptic neocortex (Keller et al., 2010), we found that ID, rather than requiring a large synchronization of neurons, can occur with relatively sparse single neuron participation (estimated at about 30% of the cells). Furthermore, a small subset of the units significantly increased or decreased their firing well before ID. Concomitant with changes in firing rate for certain neurons, at least in some patients, high-frequency oscillations at 40–120 Hz can be seen in the LFP preceding the ID and correlate closely with the changes in neuronal firing. Because interneurons are involved in the generation of high frequency oscillations through mechanisms of post-inhibition resetting of neuronal firing (Cobb et al., 1995; Ylinen et al., 1995; Le Van Quyen et al., 2008; Le Van Quyen, 2012), it is here tempting to speculate that GABA-mediated events may contribute to enhance synchronization of local epileptic networks before ID. Interestingly, emerging evidence indicates that GABA promotes epileptiform synchronization (Pavlov et al., 2013). For instance, GABA receptor-mediated inhibition can facilitate thalamocortical processes leading to the occurrence of generalized spike and wave discharges that occur during absence seizures (Danober et al., 1998). Following a similar mechanism, ID may be caused by a rebound synchronization of cells that may start firing synchronously shortly after inhibition ceases and permit the fast component of the ID. Moreover, intense synaptic activation of GABA_A_ receptors in the hippocampus can lead to a shift in GABAergic neurotransmission from inhibitory to excitatory, contributing to epileptic discharges (Kohling et al., 2000; Cohen et al., 2002). Interestingly, pre-event changes have also been seen in advance of seizures in an animal model of temporal lobe epilepsy (Bower and Buckmaster, 2008) and around seizure onset in human epilepsy (Babb and Crandall, 1976; Truccolo et al., 2011), suggesting a possible similar mechanism before seizures.

Taken together, our data suggest that ID in patients with temporal lobe epilepsy is not a simple paroxysm of hypersynchronous excitatory activity, but rather represents a heterogeneous synchronization process possibly initiated by GABAergic responses in small subsets of cells and emerging over hundreds of milliseconds before the paroxysmal discharges.

### Conflict of interest statement

The authors declare that the research was conducted in the absence of any commercial or financial relationships that could be construed as a potential conflict of interest.
